# Evaluation of clopidogrel responsiveness using the Platelet Function Analyzer-200 (PFA-200) in dogs

**DOI:** 10.3389/fvets.2025.1595147

**Published:** 2025-07-11

**Authors:** Joon-Ho Shin, Hyun-Jung Han

**Affiliations:** ^1^Department of Veterinary Emergency and Critical Care Medicine, College of Veterinary Medicine, Konkuk University, Seoul, Republic of Korea; ^2^KU Center for Animal Blood Medical Science, Konkuk University, Seoul, Republic of Korea

**Keywords:** Platelet Function Analyzer, PFA, P2Y cartridge, clopidogrel resistance, dogs

## Abstract

We aimed to evaluate the prevalence of clopidogrel resistance in hypercoagulable dogs using the Platelet Function Analyzer-200 (PFA-200) P2Y cartridge; further, we aimed to assess the utility of hematocrit (HCT), platelet count (PLT), prothrombin time (PT), activated partial thromboplastin time (aPTT), thromboelastography (TEG) parameters, and D-dimer level as indicators of clopidogrel efficacy. Forty healthy dogs underwent single measurements of P2Y closure time (CT), HCT, PLT, PT, aPTT, TEG parameters, and D-dimer levels, while thirty hypercoagulable dogs underwent two measurements of these parameters before and after clopidogrel treatment. The reference interval for P2Y CT in healthy dogs was 40.0–141.5 s, with a mean of 63.9 ± 26.82 s. Hypercoagulable dogs showed a mean baseline P2Y CT of 77.4 ± 37.6 s. Moreover, 23 (76.67%) and 7 (23.33%) showed responsiveness and resistance to the initial clopidogrel dose, respectively. The mean P2Y CT of the clopidogrel-resistant group after clopidogrel administration was 182.71 ± 78.43 s. Increasing the maintenance dose successfully overcame clopidogrel resistance in these seven dogs. Among the assessed parameters, only D-dimer levels showed a significant decrease in the clopidogrel-responder group (*p* < 0.05), suggesting its potential utility in evaluating responsiveness. In conclusion, the PFA-200 P2Y cartridge effectively detects clopidogrel resistance in dogs and can guide therapeutic adjustments such as dose escalation.

## 1 Introduction

Clopidogrel is a common antiplatelet agent in small animal practice and a second-generation thienopyridine ADP (adenosine diphosphate) receptor (P2Y12) antagonist that requires hepatic activation via cytochrome P450-dependent oxidation (1). In humans, clopidogrel response variability has been widely investigated (2, 3). Additionally, clopidogrel response variability has been demonstrated in dogs and cats (4–7). Accordingly, intended therapeutic outcomes may not be consistently achieved, and thus it is important to monitor treatment efficacy. In humans, platelet function tests identify failure to achieve the expected antiplatelet effect of clopidogrel (clopidogrel resistance) (3). In humans, clopidogrel resistance is common, with prevalence rates of 4–30% (3). Although there is limited veterinary research on clopidogrel resistance, studies have identified clopidogrel resistance in dogs using thromboelastography (TEG) with platelet mapping and multiplate, as well as in cats using light transmission aggregometry (LTA), Platelet Function Analyzer (PFA)-100, and multiplate (4–7).

**Figure 1:**
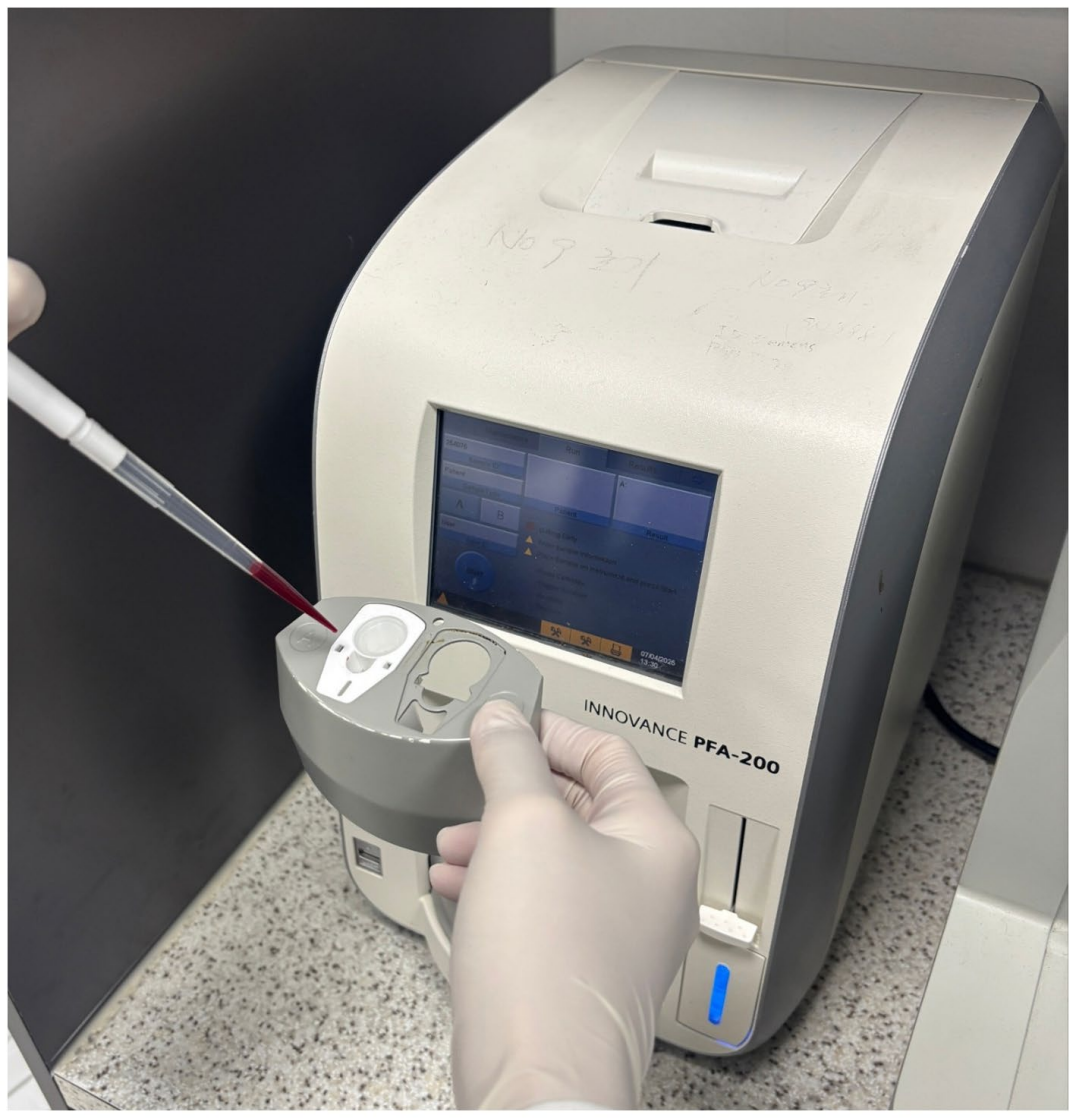
The Platelet Function Analyzer −200 device used to assess the antiplatelet effects of clopidogrel. Citrate-treated blood is dispensed into the P2Y cartridge.

The PFA-100/200 system (Siemens Healthcare, Germany) is a cartridge-based point-of-care assay that evaluates platelet function in high-shear-stress conditions by measuring the closure time (CT) of a membrane aperture (Figure 1). The PFA P2Y cartridge can assess the antiplatelet effects of clopidogrel. Although the current gold standard for platelet function testing is LTA, the PFA is simpler and faster, and thus more suitable for in-hospital testing (8). Additionally, compared to TEG with platelet mapping, the PFA is more cost-effective and offers quicker assessment.

We aimed to establish a reference interval (RI) for P2Y CT in healthy dogs and observe changes in P2Y CT following clopidogrel administration in order to assess clopidogrel responsiveness in dogs. Additionally, we evaluated the utility of conventional coagulation-related markers, including hematocrit (HCT), platelet count (PLT), prothrombin time (PT), activated partial thromboplastin time (aPTT), TEG parameters, and D-dimer level, as indicators of clopidogrel efficacy.

## 2 Materials and methods

### 2.1 Animals

This prospective study included hypercoagulable dogs diagnosed via TEG at Konkuk Veterinary Medical Teaching Hospital and healthy donor dogs from the KU I’M DOgNOR Blood Donation Center. Ethical approval was granted by the Institutional Animal Care and Use Committee at Konkuk University, under approval number [KU23191, KU24249]. Dogs exhibiting hypercoagulability, defined by TEG parameters with a maximum amplitude (MA) > 69 (9, 10) and a coagulation index (CI) > 4 (11), were included. Exclusion criteria included the use of drugs that could potentially interfere with P2Y CT, including antiplatelet agents such as clopidogrel, aspirin, cilostazol, and tirofiban; anticoagulants such as heparin, rivaroxaban, and warfarin; and thrombolytic agents such as streptokinase, as well as a history of bleeding disorders or a platelet count of <100 × 10^9^/L. The healthy group comprised dogs without underlying diseases and normal findings in complete blood count, electrolytes (sodium, chloride, potassium), serum chemistry (albumin, alkaline phosphatase, alanine aminotransferase, blood urea nitrogen, creatinine, glucose, total protein), and coagulation tests (PT, aPTT).

### 2.2 Study design

Healthy dogs underwent TEG, PFA P2Y, and D-dimer tests. Hypercoagulable dogs underwent tests for P2Y CT, complete blood count, PT, aPTT, and D-dimer levels at diagnosis. Subsequently, they received 10 mg/kg clopidogrel (loading dose), followed by 2 mg/kg once daily for ≥4 days. Blood samples were obtained 5–7 days after initiation and 12–14 h following the last dose for re-assessments of the aforementioned parameters.

### 2.3 Blood collection

To minimize mechanical stress on platelets during sample collection, blood was drawn from the jugular vein using needles no smaller than 21 gauge. After collection, the needle was removed from the syringe before gently transferring the blood into ethylenediaminetetraacetic acid and citrate tubes. We collected 0.3 mL of blood into tubes containing ethylenediaminetetraacetic acid for complete blood count analysis, and 2.7 mL of blood into 3.2% sodium citrate tubes using an exact 1:9 ratio of anticoagulant to blood for TEG, PFA P2Y, PT, aPTT, and D-dimer testing.

### 2.4 TEG test procedure

Whole blood collected in 3.2% sodium citrate was gently mixed and then rested at room temperature (20–25°C) for 15 min in accordance with the manufacturer’s instructions. One milliliter of citrated whole blood was transferred into a proprietary kaolin tube (Haemonetics^®^, Braintree, MA, USA) containing kaolin, phospholipids, and stabilizers. From this, 340 μL was placed into a TEG cup and recalcified with 20 μL of 200 mM calcium chloride. Standard TEG parameters (reaction time [R], alpha angle [*α*], coagulation time [K], and MA) were recorded. The CI was calculated as follows (12):

CI=0.1227(R)+0.0092(K)+0.1655(MA)–0.0241(α)–5.0220

### 2.5 PFA P2Y test procedure

As with the TEG assay, whole blood collected in 3.2% sodium citrate was gently mixed and then rested without agitation at room temperature (20–25°C) for 15 min, in accordance with the manufacturer’s instructions. Using the PFA-200, 800–900 μL of citrated whole blood was pipetted into a prewarmed P2Y test cartridge. P2Y CT was measured with a maximum threshold of 300 s; with values exceeding 300 s being reported as non-closure and recorded as 300 s. Dogs exhibiting non-closure in P2Y CT after clopidogrel administration were classified as the clopidogrel-responder group, whereas those with a P2Y CT of less than 300 s were classified as the clopidogrel-resistant group. Samples were tested within 1 h after venipuncture.

### 2.6 Other blood test procedures

HCT and PLT were measured using an automated hematology analyzer (IDEXX ProCyte Dx, IDEXX Laboratories, Inc., Westbrook, ME, USA). PT and aPTT were measured with a coagulation analyzer (IDEXX Coag Dx, IDEXX Laboratories, Inc., Westbrook, ME, USA). D-dimer levels were determined by a fluorescent immunoassay (Vet Chroma™, ANIVET Diagnostic Inc., Chuncheon, Kangwon, South Korea), with a detection range of 50–10,000 ng/mL. Values <50 ng/mL and >10,000 ng/mL were recorded as 50 ng/mL and 10,000 ng/mL, respectively.

### 2.7 Statistical analysis

All statistical analyses were performed using SPSS software (version 29.0, IBM Corp., Armonk, NY, USA). Statistical significance was defined as *p* < 0.05. Normality was assessed by the Shapiro–Wilk test. For normally distributed data, independent samples *t*-tests were used for between-group comparisons and paired t-tests for pre- and post-treatment comparisons. For non-normally distributed data, the Mann–Whitney U and Wilcoxon signed-rank tests were used. Reference Value Advisor v2.1 was used to calculate the RI for P2Y CT (13). Although robust methods are recommended by the American Society for Veterinary Clinical Pathology (ASVCP) guidelines (14), their application in this data set yielded unreliable estimates, likely due to a limited sample size. Therefore, a nonparametric percentile method was used as a more reliable alternative. Outliers were evaluated using Tukey’s method, which defines outliers as values falling below the first quartile minus 1.5 times the interquartile range (IQR) or above the third quartile plus 1.5 times the IQR. All identified outliers were assessed for potential analytical or clinical exclusion in accordance with ASVCP guidelines.

## 3 Results

### 3.1 Animals

A total of 70 dogs were included in this study, consisting of 40 healthy dogs and 30 dogs diagnosed with hypercoagulability. The characteristics of the dogs in both the healthy control and hypercoagulable groups, including sex, breed, weight, and age are presented in an electronic spreadsheet database (Supplementary Table 1). The hypercoagulable dogs had a variety of underlying conditions, such as immune-mediated diseases, inflammation, and hemodynamic compromise. The medical information, including the diagnoses, was organized in the Supplementary Table 1.

### 3.2 PFA P2Y test results

**Figure 2:**
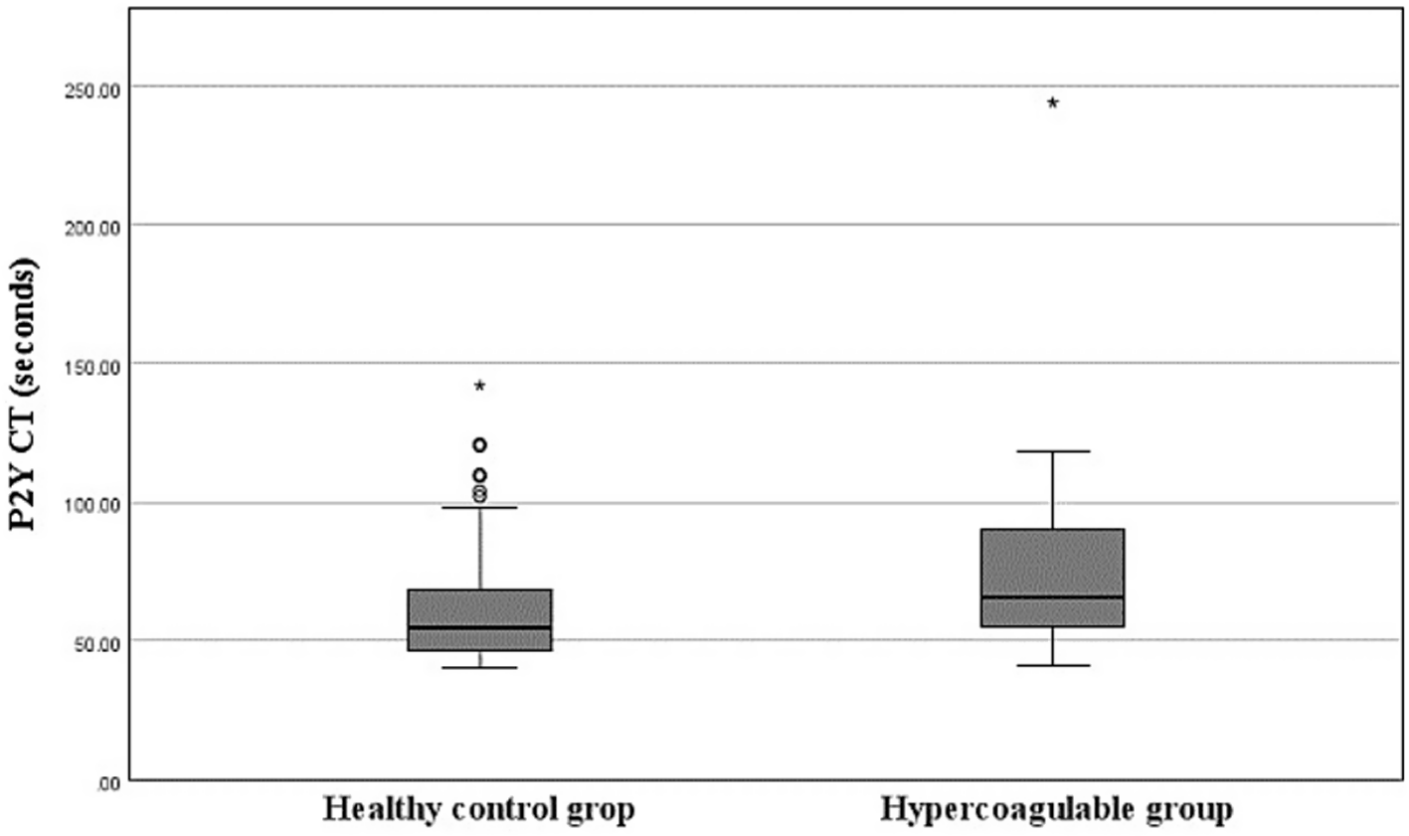
Box-and-Whisker plots of P2Y closure time (CT) in the healthy and hypercoagulable groups. The boxes indicate the interquartile ranges (25^th^–75^th^ percentile) and lines within the boxes indicate the median values. Whiskers indicate either 1.5 times the interquartile range or the range limit for the data, whichever is less. Circles represent mild outliers, which are values exceeding 1.5 times the interquartile range below the 25th percentile or above the 75th percentile. Asterisks represent extreme outliers, which are values exceeding three times the interquartile range beyond these percentiles. The hypercoagulable group shows prolonged P2Y CT compared to the healthy group (*p* < 0.05, Mann–Whitney U test).

The mean P2Y CT in the healthy group was 63.9 ± 26.82 s (minimum, 40 s; maximum, 142 s), which was shorter than in the hypercoagulable group (77.37 ± 37.61 s; minimum, 41 s; maximum, 244 s; *p* < 0.05; Figure 2). The RI for P2Y CT in the healthy group was determined to be 40.0–141.5 s using a nonparametric percentile method. The 90% confidence intervals for the lower and upper RI limits, calculated by bootstrap resampling, were 40.0–41.0 s and 119.6–142.0 s, respectively. Outlier analysis using Tukey’s method identified one value exceeding the upper fence, but this value was retained in the final RI as no clinical or analytical justification for exclusion was identified.

In the hypercoagulable group, 23 (76.67%) and 7 (23.33%) dogs were classified as the clopidogrel-responder and clopidogrel-resistant groups, respectively (Table 1). In the clopidogrel-responder group, the mean P2Y CT significantly increased from 77.48 ± 41.67 s (41.00–244.00) to >300 s (*p* < 0.05); similarly, it increased from 77.00 ± 21.63 s (55.00–118.00) s to 182.71 ± 78.43 s (70.00–273.00) in the clopidogrel-resistant group (*p* < 0.05).

**Table 1:** Comparison of P2Y closure times before and after treatment with clopidogrel in clopidogrel-responder and clopidogrel-resistance groups.

Variables	Clopidogrel responder (n = 23)			Clopidogrel-resistance (*n* = 7)		
	Before	After	*p-*value	Before	After	*p*-value
P2Y CT (s)	77.48 ± 41.67 (41.00–244.00)	300.00 ± 0.00 (300.00–300)	^*^ < 0.001	77.00 ± 21.63 (55.00–118.00)	182.71 ± 78.43 (70.00–273.00)	^*^0.018

Mean ± SD (range), CT; closure time. ^*^p-value<0.05 indicates significance.

**Figure 3:**
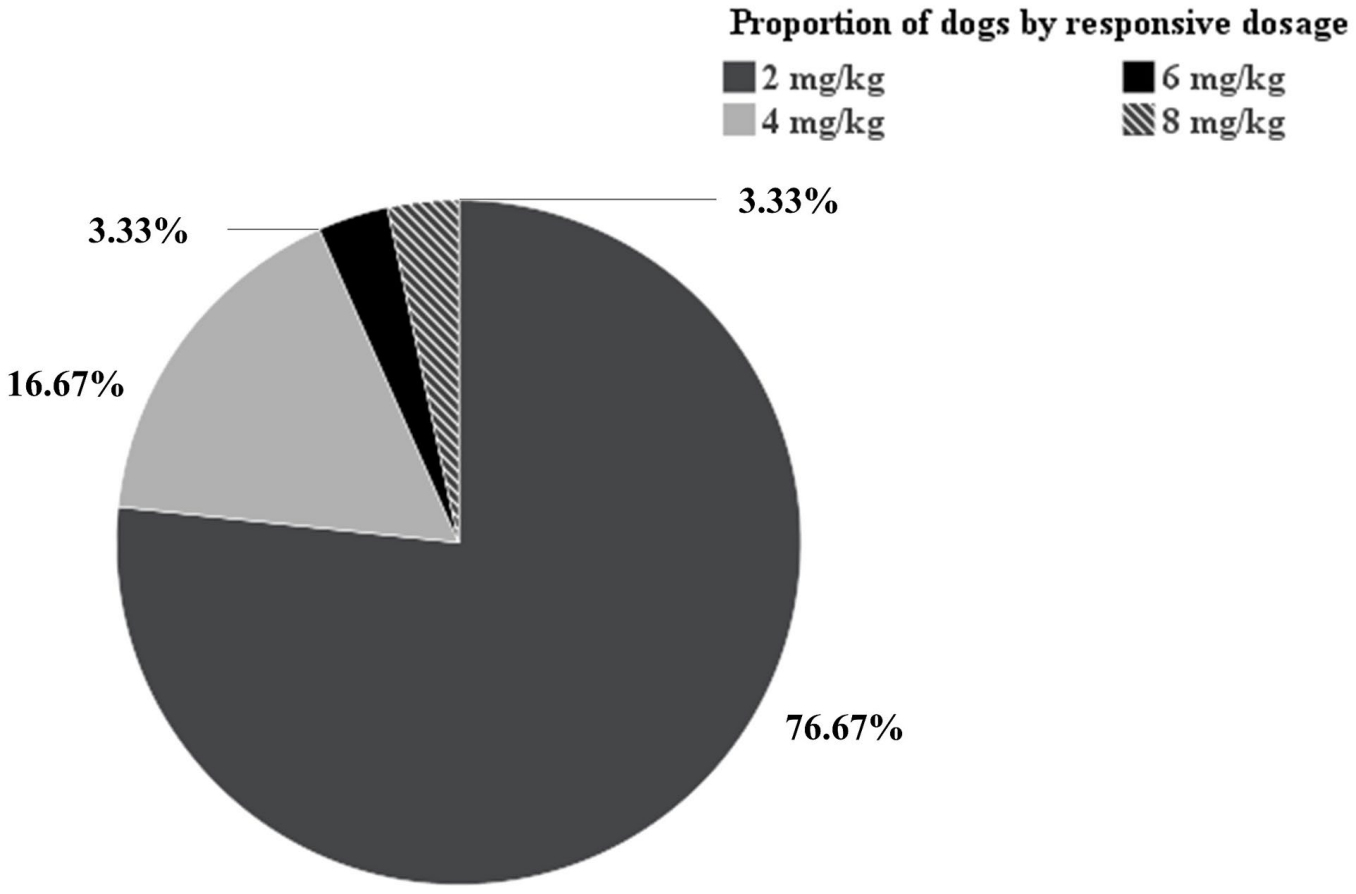
Pie chart of proportion of dogs by responsive dosage. Among the 30 dogs in the hypercoagulable group, 76.67, 16.67, 3.33, 3.33% of the dogs showed clopidogrel responsiveness at 2 mg/kg, 4 mg/kg, 6 mg/kg, and 8 mg/kg once daily, respectively.

In the clopidogrel-resistant group, the maintenance dose was initially increased from 2 mg/kg to 4 mg/kg, which was administered for 5 days, followed by P2Y CT remeasurement. If the P2Y CT remained <300 s, the dose was further increased to 6 mg/kg, followed by to 8 mg/kg, until the P2Y CT exceeded 300 s. In this group, five, one, and one dog achieved a P2Y CT > 300 s at 4 mg/kg, 6 mg/kg, and 8 mg/kg once daily, respectively. In the hypercoagulable group, 76.67, 16.67, 3.33, 3.33% of the dogs showed clopidogrel responsiveness at 2 mg/kg, 4 mg/kg, 6 mg/kg, and 8 mg/kg once daily, respectively (Figure 3).

### 3.3 Hematological (HCT, PLT) and coagulation (PT, aPTT, D-dimer, R, K, *α*, MA, CI) test results

In the healthy group, the mean HCT, PLT count, PT, and aPTT were 48.63 ± 4.55% (40.20–60.80), 207.95 ± 42.61 K/μL (150.00–300.00), 14.58 ± 1.71 s (12.00–17.00), and 82.68 ± 7.12 s (72.00–101.00), respectively. The mean D-dimer levels was 474.26 ± 1110.54 ng/mL (50.00–6634.98). For TEG parameters, the mean R, K, *α*, MA, and CI were 2.38 ± 0.99 min (0.70–4.90), 4.64 ± 2.78 min (0.80–13.50), 53.54 ± 14.29 (22.80–81.70), 51.88 ± 6.72 mm (35.80–62.50), and 2.60 ± 0.86 (0.94–4.00), respectively.

Table 2 presents the hematological and coagulation test results. In the clopidogrel-responder group (*n* = 23), mean HCT did not significantly differ, changing from 35.06 ± 8.28% (22.60–57.70) to 36.01 ± 6.04% (25.90–45.80) (*p* = 0.456) (Table 2). The mean PLT significantly increased from 356.30 ± 142.73 K/μL (100.00–591.00) to 450.17 ± 184.52 K/μL (227.00–792.00) (*p* < 0.05). PT remained stable, with values of 13.48 ± 1.12 s (11.00–16.00) before and 13.09 ± 1.50 s (11.00–16.00) after treatment (*p* = 0.441). Mean aPTT significantly decreased from 96.17 ± 10.28 s (78.00–119.00) to 88.09 ± 6.98 s (76.00–100.00) (*p* < 0.05). D-dimer levels showed a significant reduction from 2476.95 ± 3185.40 ng/mL (50.00–10000.00) to 775.29 ± 880.31 ng/mL (50.00–3786.00) (*p* < 0.05). For TEG parameters, the mean R did not significantly differ, changing from 3.14 ± 1.19 min (1.60–6.40) to 3.27 ± 1.29 min (1.50–5.80) (*p* = 0.681), K showed no meaningful difference, changing from 1.14 ± 0.48 min (0.80–2.70) to 1.43 ± 0.94 min (0.80–3.70) (*p* = 0.38), *α* did not significantly differ, changing from 73.30 ± 8.02 (43.60–82.70) to 70.7 ± 9.85° (48.20–81.40) (*p* = 0.336), MA did not change significantly, changing from 77.59 ± 3.87 mm (70.10–84.20) to 74.52 ± 7.24 mm (55.30–83.70) (*p* = 0.176), and CI did not differ significantly, changing from 6.37 ± 0.78 (4.2–7.33) to 6.02 ± 1.02 (3.38–7.35) (*p* = 0.069).

**Table 2:** Hematological and coagulation test results before and after clopidogrel administration in the clopidogrel-responder and clopidogrel-resistance groups.

Variables	Reference interval	Clopidogrel responder			Clopidogrel-resistance		
		Before	After	*p-*value	Before	After	*p-*value
Hematocrit (%)	37.3–61.7	35.06 ± 8.28 (22.60–57.70)	36.01 ± 6.04 (25.90–45.80)	0.456	33.89 ± 8.79 (17.60–41.80)	33.43 ± 11.37 (21.40–50.80)	1.00
Platelet (K/μL)	148–484	356.30 ± 142.73 (100.00–591.00)	450.17 ± 184.52 (227.00–792.00)	^*^0.01	443.57 ± 151.22 (308.00–720.00)	429.86 ± 148.43 (259.00–683.00)	0.612
PT (s)	11–17	13.48 ± 1.12 (11.00–16.00)	13.09 ± 1.50 (11.00–16.00)	0.441	13.57 ± 2.07 (11.00–16.00)	13.86 ± 1.86 (13.00–18.00)	1.00
aPTT (s)	72–102	96.17 ± 10.28 (78.00–119.00)	88.09 ± 6.98 (76.00–100.00)	^*^0.011	95.00 ± 12.60 (77.00–112.00)	91.86 ± 16.04 (72.00–113.00)	0.499
D-dimer (ng/mL)	< 250	2476.95 ± 3185.40 (50.00–10000.00)	775.29 ± 880.31 (50.00–3786.00)	^*^0.01	681.56 ± 523.00 (127.70–1780.21)	782.23 ± 810.40 (62.60–1925.10)	0.735
R (minute)	1.8–8.6	3.14 ± 1.19 (1.60–6.40)	3.27 ± 1.29 (1.50–5.80)	0.681	3.69 ± 0.96 (2.80–5.20)	2.89 ± 1.21 (1.40–4.70)	0.116
K (minute)	1.3–5.7	1.14 ± 0.48 (0.80–2.70)	1.43 ± 0.94 (0.80–3.70)	0.38	1.21 ± 0.47 (0.80–2.10)	1.34 ± 0.71 (0.80–2.70)	1.00
α (°)	36.9–74.6	73.30 ± 8.02 (43.60–82.70)	70.70 ± 9.85 (48.20–81.40)	0.336	72.13 ± 6.90 (59.70–78.80)	72.70 ± 8.00 (58.00–79.90)	0.735
MA (mm)	42.9–67.9	77.59 ± 3.87 (70.10–84.20)	74.52 ± 7.24 (55.30–83.70)	0.176	78.49 ± 6.07 (70.40–85.10)	75.36 ± 8.75 (62.40–84.60)	0.352
CI	-4–4	6.37 ± 0.78 (4.20–7.33)	6.02 ± 1.02 (3.38–7.35)	0.069	6.19 ± 1.19 (4.24–7.58)	6.30 ± 1.13 (4.24–7.64)	0.109

Mean ± SD (range). HCT; hematocrit, PLT; platelet count, PT; prothrombin time, aPTT; activated partial thromboplastin time, R; reaction time, K; coagulation time, α; alpha angle, MA; maximum amplitude, CI; coagulation index. *p-value<0.05 indicates significance.

In the clopidogrel-resistant group (*n* = 7), the mean HCT showed no meaningful difference, changing from 33.89 ± 8.79% (17.60–41.80) to 33.43 ± 11.37% (21.40–50.80) (*p* = 1.00) (Table 2). The mean PLT did not significantly differ, changing from 443.57 ± 151.22 K/μL (308.00–720.00) to 429.86 ± 148.43 K/μL (259.00–683.00) (*p* = 0.612). PT did not change significantly, changing from 13.57 ± 2.07 s (11.00–16.00) to 13.86 ± 1.86 s (13.00–18.00) (*p* = 1.00). Mean aPTT did not differ significantly, changing from 95.00 ± 12.60 s (77.00–112.00) to 91.86 ± 16.04 s (72.00–113.00) (*p* = 0.499). D-dimer levels did not significantly differ, changing from 681.56 ± 523.00 ng/mL (127.70–1780.21) to 782.23 ± 810.40 ng/mL (62.60–1925.10) (*p* = 0.735). For TEG parameters, mean R showed no meaningful difference, changing from 3.69 ± 0.96 min (2.80–5.20) to 22.89 ± 1.21 min (1.40–4.70) (*p* = 0.116), K did not significantly differ, changing from 1.21 ± 0.47 min (0.80–2.10) to 1.34 ± 0.71 min (0.80–2.70) after (*p* = 1.00), α did not change significantly, changing from 72.13 ± 6.9 (59.70–78.80) to 72.70 ± 8.00 (58.00–79.90) (*p* = 0.735), MA did not differ significantly, changing from 78.49 ± 6.07 mm (70.40–85.10) to 75.36 ± 8.75 mm (62.40–84.60) (*p* = 0.352), and CI did not significantly differ, changing from 6.19 ± 1.19 (4.24–7.58) to 6.30 ± 1.13 (4.24–7.64) (*p* = 0.109).

## 4 Discussion

Antiplatelets have been traditionally used to prevent arterial thrombosis, with emerging evidence suggesting platelets also contribute to venous thrombosis (15–18). In humans, antiplatelet agents have demonstrated efficacy in preventing venous thrombosis (19–21), suggesting that the application of clopidogrel in veterinary medicine can be expanded. Additionally, advancements in coagulation tests to facilitate hypercoagulability diagnosis (22, 23), as well as advances in interventional cardiology and open-heart surgery in veterinary medicine, have led to an increase in clopidogrel prescriptions (24, 25).

Clopidogrel resistance is defined as insufficient platelet inhibition *in vitro* following drug administration (3). In humans, the CYP2C19 gene, which is essential for clopidogrel metabolism, exhibits various polymorphisms that lead to significant variability in enzyme activity, which is the primary cause of clopidogrel resistance (26–28). Other known mechanisms of clopidogrel resistance in humans include differences in intestinal absorption, genetic polymorphisms of ABC1 as well as GpIIb-IIIa, GPIa-IIa and P2Y12 receptors, and clinical factors such as diabetes and overweightness (29). In cats, mutations in the P2RY1 gene, one of the platelet ADP receptor genes, excessively activates the P2Y1 receptor in response to ADP, contributing to clopidogrel resistance (30). The mechanisms underlying clopidogrel resistance in dogs remains unclear. Nonetheless, clopidogrel resistance in dogs is considered to involve genetic polymorphisms in either platelet ADP receptor genes (P2RY1, P2RY12) or the cytochrome P450 enzyme gene.

In humans, clopidogrel resistance is clinically associated with an increased risk of thrombosis (2, 31, 32). Although therapeutic strategies for clopidogrel resistance remain inconclusive in humans, increasing the loading dose, using a higher maintenance dose, or switching to alternative thienopyridines such as ticlopidine or prasugrel, may enhance platelet inhibition in clopidogrel-resistant patients (29, 33). In veterinary medicine, there remains scarce research on treatment strategies related to clopidogrel resistance. Therefore, it is important to identify patients with inadequate responses to clopidogrel and implement alternative therapeutic strategies in order to improve prognosis and survival in veterinary patients.

There remain no established standards for evaluating the efficacy of antiplatelet therapy in dogs, which is further complicated by inconsistencies across platelet function tests and the lack of an established RI for treatment monitoring. Platelet function tests measure the inhibition of ADP-induced platelet aggregation to evaluate the efficacy and resistance of clopidogrel; however, the methods differ across devices (8). LTA and turbidimetric-based optical detection systems both assess platelet aggregation by detecting changes in light transmission, using platelet-rich plasma and whole blood, respectively. Another method, electrical aggregometry, assesses platelet aggregation by measuring changes in electrical impedance as platelets adhere to electrodes in whole blood. TEG with platelet mapping measures platelet aggregation and thrombus formation in real-time, indirectly confirming clopidogrel inhibition by observing a decrease in clot strength. Flow cytometry evaluates the expression and activation status of platelet surface receptors to assess the effectiveness of clopidogrel-mediated P2Y12 receptor inhibition. Furthermore, the PFA P2Y test is a useful tool for assessing the antiplatelet effects of clopidogrel. This cartridge-based point-of-care assay involves aspirating whole blood through a small aperture (150-μm diameter) in a membrane coated with 20 μg of ADP, 5 ng of prostaglandin E1, and 459 μg of calcium chloride. Under high shear conditions, these platelet activators induce platelet plug formation, eventually occluding the aperture, with the time until full occlusion being measured.

LTA is considered the gold standard for evaluating the antiplatelet effect of clopidogrel. However, ADP-induced LTA may be slightly inappropriate for evaluating clopidogrel’s effect since ADP can induce platelet aggregation through both the P2Y12 and P2Y1 receptors, meaning that using ADP alone may lack the specificity required to accurately assess clopidogrel’s effect (3, 34, 35). LTA requires larger sample volumes and a high level of technical expertise, which impedes its practicability in routine clinical settings, especially in veterinary medicine. Additionally, electrical aggregometry and flow cytometry also require a high level of technical expertise, while TEG with platelet mapping is relatively expensive and time-consuming.

Contrastingly, the PFA is simpler and only requires small blood volumes; therefore, it is widely used in clinical practice. The PFA has three cartridge types, including the collagen/epinephrine cartridge (C/Epi), which uses collagen and epinephrine as platelet activators; the collagen/adenosine diphosphate cartridge (C/ADP), which uses collagen and adenosine diphosphate; and the P2Y cartridge, which is coated with prostaglandin E1. In humans, both C/Epi and C/ADP cartridges are used to evaluate platelet function, showing particular sensitivity to von Willebrand disease, and the C/Epi cartridge is especially sensitive to aspirin therapy (36). Both cartridges are useful for evaluating primary hemostasis disorders in dogs (37); however, the role of epinephrine remains unclear in canine platelet aggregation, which differs from humans (38, 39). Accordingly, the use of the C/Epi cartridge in assessing canine platelet function remains controversial. Contrastingly, ADP is highly sensitive to platelet aggregation in dogs, making the C/ADP cartridge useful for evaluating platelet function (40–42). Specifically, P2Y cartridge is considered more suitable for evaluating clopidogrel efficacy since it inhibits P2Y1-mediated aggregation using prostaglandin E1 in the cartridge, facilitating more specific evaluation of P2Y12-mediated aggregation, which is directly influenced by clopidogrel. In cats, the P2Y cartridge is more sensitive and effective than the C/ADP cartridge in evaluating clopidogrel efficacy, expanding the potential applications of the PFA in veterinary medicine (43). We performed the PFA P2Y test using a relatively small volume of whole blood (approximately 800 μL), making the testing procedure simple and quick (5–10 min). These factors contribute to the practicality and ease of applying this method in veterinary clinical settings. Consequently, via this clinical utility, we established the RI for P2Y CT using P2Y cartridge and observed that some P2Y CT parameters were significantly longer in the hypercoagulable group than in the healthy group. Further, 76.67% of the clopidogrel-administered dogs showed clopidogrel responsiveness, with a P2Y CT > 300 s.

Notably, the PFA P2Y test has several limitations, including a capped measurement limit of 300 s for CT, beyond which values are not recorded, and the influence by various hematologic factors. If a patient’s P2Y CT prior to clopidogrel administration already exceeds 300 s, it may be impossible to use the PFA P2Y test to monitor treatment responsiveness. Additionally, human studies on C/Epi and C/ADP cartridges have showed that significant reductions in PLT or HCT, lower von Willebrand factor levels, and the use of higher-concentration sodium citrate tubes all led to prolonged CT (44–46). Furthermore, these factors may similarly affect the P2Y cartridge, making it unsuitable for dogs with low PLT, HCT, or von Willebrand factor levels. In our study, all blood samples for the PFA P2Y test were uniformly treated with 3.2% sodium citrate, which eliminated the effects of variations in sodium citrate concentration. Therefore, the relatively low HCT in the hypercoagulable group may have contributed to the prolonged P2Y CT. Furthermore, one hypercoagulable dog, with a P2Y CT of 244 s outside the normal reference range, presented with thrombocytopenia (100 × 10^9^/L). This suggests that the low platelet count may have contributed to the prolonged CT.

In dogs, a P2Y CT < 300 s after clopidogrel administration is considered indicative of clopidogrel resistance (47). The rate of clopidogrel resistance in our study was 23.33%, which is consistent with a previously reported value of 25% (4) and comparable to that in humans (4–30%) (3), although its clinical implications may differ. Unlike in humans, where clopidogrel is administered at a fixed dose of 300 mg on the first day, followed by 75 mg/day, dogs are typically dosed based on body weight. Additionally, the broader dosing range for clopidogrel in dogs (1–4 mg/kg) (48–52) and the variability in the use of a loading dose impedes determination of an optimal personalized dose without inducing resistance or causing adverse effects. These challenges in dogs emphasize the need for monitoring clopidogrel efficacy.

Our findings showed that the effective clopidogrel dosage varies across dogs, highlighting the importance of confirming clopidogrel efficacy. Furthermore, increasing the maintenance dose helped overcome resistance. In this study, we implemented a personalized antiplatelet therapy inspired by therapeutic strategies from human medicine for managing clopidogrel resistance (33). To date, the highest maintenance dose of clopidogrel reported in the veterinary literature is 4 mg/kg (48). Therefore, we established 8 mg/kg, which is twice that amount, as the maximum maintenance dose in our protocol. To minimize the risk of adverse effects associated with high-dose escalation, an intermediate step of 6 mg/kg was incorporated. For dogs receiving doses exceeding the standard maintenance regimen, consent was obtained from the owners after providing a comprehensive explanation of the rationale, potential benefits, and possible adverse effects of the proposed treatment. Safety monitoring consisted of complete blood count assessments and thorough clinical evaluations for signs of bleeding, including petechiae, ecchymosis, epistaxis, melena, and hematochezia. These evaluations were conducted on days 2 and 5 following clopidogrel administration. No adverse events were observed in any dog throughout the study period. All dogs exhibiting resistance responded when the dose was increased to a maximum of 8 mg/kg once daily. However, for dogs that continue to exhibit resistance despite this dose adjustment, other thienopyridine therapies, similar to those used in humans, could be an alternative. Nonetheless, further studies are warranted to validate these approaches in veterinary medicine.

Among the conventional coagulation markers, only PLT, aPTT, and D-dimer levels showed significant pre-post differences in the clopidogrel-responder group. Following clopidogrel administration, the increase in PLT and shortening of aPTT is suggestive of enhanced coagulation. However, no significant changes were observed in TEG parameters, and P2Y CT was prolonged after clopidogrel administration. Therefore, the increase in PLT and shortening of aPTT were not considered significant risk factors for hypercoagulability or increased thrombus formation following clopidogrel administration. Although D-dimer levels cannot be considered a specific indicator of thrombus formation, a decrease in D-dimer level reflects reduced thrombin generation and fibrin turnover, which is associated with a decreased risk of thrombus-related cardiovascular events in humans (53). Consequently, D-dimer levels only significantly decreased in the clopidogrel-responder group, suggesting that clopidogrel may have contributed to reducing blood clot formation. Among the conventional markers, D-dimer could be useful in evaluating clopidogrel responsiveness.

This study has several limitations. First, the relatively small sample size, particularly in the clopidogrel-resistant group, may have impeded the statistical robustness and the generalizability of the findings. Anemia, characterized by low HCT, may influence the accuracy of CT measurements. A previous study in human medicine has reported that HCT levels below 25% can result in CT prolongation (54). In the present study, 18 of the 30 dogs in the hypercoagulable group were anemic (HCT < 36%), yet 17 of these had P2Y CT values within the established reference interval (40.0–141.5 s). Given that the primary objective of this study was to evaluate within-individual pre- and post-treatment changes, these cases were retained in the analysis. Furthermore, a prior canine study has shown that the extent of CT prolongation associated with low HCT is relatively modest (55). Nonetheless, in dogs with moderate to severe anemia (HCT < 30%), the potential impact of HCT on P2Y CT values should be recognized as a limitation of this study, warranting cautious interpretation and further investigation. The healthy group mainly comprised large-breed dogs, while the hypercoagulable group was composed primarily of small-breed dogs, introducing potential breed-related variability. Additionally, the underlying causes of hypercoagulability and their treatment strategies differed across the dogs, which may have influenced the results. However, in the hypercoagulable group, no dogs were receiving any antiplatelet or anticoagulant therapy except for clopidogrel.

Another consideration is that the mean D-dimer concentration in the healthy group was unexpectedly high. The median D-dimer concentration in this group was 152.29 ng/mL, indicating that most healthy dogs had physiologically normal levels. However, three dogs exhibited markedly elevated values of 2199.84, 2287.67, and 6634.98 ng/mL, which significantly influenced the group mean. Vigorous physical activity prior to blood sampling, such as playing fetch, may have contributed to this finding, as reported in human studies demonstrating exercise-induced increases in D-dimer levels (56). While this variability may influence interpretation, the PFA P2Y cartridge is less likely to be affected by such factors, as it specifically evaluates the P2Y12 receptor pathway and minimizes the impact of non-specific physiological changes. Importantly, our analysis focused on within-individual changes, and a significant reduction in D-dimer levels was still observed in responders. Nevertheless, as D-dimer is a non-specific marker, reliance on absolute values should be avoided, and this should be acknowledged as a potential limitation in interpreting the study findings. Finally, although we identified clopidogrel resistance in some dogs, we did not evaluate long-term clinical outcomes such as thromboembolic events or bleeding complications. Given the role of genetic factors, such as CYP2C19 and P2Y12 receptor polymorphisms, in clopidogrel resistance in humans, future studies investigating genetic variability in dogs are warranted to improve our understanding of the underlying mechanisms of clopidogrel resistance and to optimize antiplatelet therapy in this species.

## 5 Conclusion

Our findings confirmed individual variability in response to clopidogrel, emphasizing the importance of monitoring clopidogrel efficacy in dogs. The PFA P2Y test effectively assessed clopidogrel efficacy, and thus is a highly useful tool for veterinarians to objectively monitor clopidogrel responsiveness in dogs and inform therapeutic strategies.

## Data Availability

The original contributions presented in the study are included in the article/Supplementary material, further inquiries can be directed to the corresponding author.
