# Supplementary material for: Evaluation of clopidogrel responsiveness using the Platelet Function Analyzer-200 (PFA-200) in dogs
Source: Front Vet Sci. 2025 Jul 11;12:1595147. doi: 10.3389/fvets.2025.1595147 (PMC12292019; doi:10.3389/fvets.2025.1595147)
Supplement: Supplementary file 1 [file Table_1.docx]

Supplementary Material

**Table 1.** Demographic and clinical characteristics of study dogs

| Age | Gender | Body weight | Diagnosis | Surgery | HCT (%) | PLT (K/μL) | PT(seconds) | aPTT(seconds) | D-dimer (ng/mL) | R time (min) | K time (min) | α (degree) | MA (mm) | CI | P2Y CT |  |
| --- | --- | --- | --- | --- | --- | --- | --- | --- | --- | --- | --- | --- | --- | --- | --- | --- |
| 5y | SF | 27.2 | Healthy |  | 45.4 | 190 | 12 | 83 | 62.98 | 2.2 | 3.3 | 60.8 | 59.4 | 3.64372 | 102 |  |
| 2y | SF | 30.2 |  |  | 42.1 | 151 | 14 | 72 | 322.54 | 1.7 | 3.8 | 57.5 | 54.1 | 2.78935 | 53 |  |
| 3y | SF | 33.9 |  |  | 43.5 | 295 | 14 | 76 | 6634.98 | 1.7 | 2.9 | 56.7 | 56.1 | 3.13135 | 98 |  |
| 3y | SF | 30.4 |  |  | 40.2 | 157 | 16 | 84 | 437.93 | 2.2 | 4.2 | 48.5 | 52 | 2.72373 | 40 |  |
| 4y | CM | 28 |  |  | 42 | 259 | 12 | 82 | 120.01 | 4.9 | 4.7 | 58.3 | 57.8 | 3.78334 | 44 |  |
| 5y | CM | 27 |  |  | 49.3 | 173 | 17 | 86 | 460 | 2.3 | 4.4 | 50.5 | 48.4 | 2.09384 | 58 |  |
| 4y | CM | 28 |  |  | 43 | 230 | 16 | 80 | 230.87 | 1.8 | 4.8 | 51.7 | 51.1 | 2.4541 | 41 |  |
| 3y | CM | 43.7 |  |  | 45.9 | 250 | 14 | 81 | 212.07 | 2 | 4.8 | 51.4 | 50.6 | 2.40312 | 49 |  |
| 3y | CM | 38.7 |  |  | 51.1 | 250 | 14 | 87 | 90.16 | 3 | 5.2 | 40 | 47.4 | 2.27464 | 56 |  |
| 3y | CM | 33 |  |  | 51.7 | 150 | 14 | 82 | 437.55 | 4.2 | 8.2 | 30.3 | 46.2 | 2.48465 | 46 |  |
| 1y | CM | 28 |  |  | 47.3 | 185 | 14 | 81 | 93.2 | 3.8 | 12 | 22.8 | 41.4 | 1.85688 | 142 |  |
| 2y | CM | 35.7 |  |  | 46.9 | 174 | 15 | 99 | 64.35 | 4.2 | 13.5 | 39.8 | 43.1 | 1.79141 | 47 |  |
| 3y | CM | 40.8 |  |  | 43.9 | 195 | 14 | 83 | 121.97 | 2.6 | 2.9 | 56.8 | 57.7 | 3.50417 | 45 |  |
| 4y | CM | 45 |  |  | 53.9 | 224 | 14 | 101 | 240.55 | 4.5 | 9.8 | 25 | 35.8 | 0.94271 | 40 |  |
| 4y | IF | 36 |  |  | 46.5 | 215 | 12 | 91 | 150.75 | 2.8 | 4.1 | 46.9 | 50.4 | 2.57019 | 104 |  |
| 3y | CM | 37.5 |  |  | 46.4 | 183 | 14 | 73 | 156.99 | 2.2 | 3.4 | 51.5 | 53.5 | 2.89232 | 50 |  |
| 2y | SF | 36.3 |  |  | 45.4 | 235 | 12 | 83 | 341.71 | 2.2 | 4.5 | 39.7 | 55.5 | 3.51782 | 47 |  |
| 8y | CM | 34 |  |  | 60.8 | 152 | 14 | 82 | 2287.67 | 2.7 | 7.1 | 45.5 | 48.3 | 2.27171 | 47 |  |
| 4y | CM | 31 |  |  | 51.5 | 232 | 17 | 88 | <50 | 1.1 | 3 | 70.1 | 50.3 | 1.77581 | 59 |  |
| 4y | SF | 26 |  |  | 48.2 | 297 | 17 | 74 | 94.78 | 1.4 | 1.2 | 74.1 | 62.5 | 3.71876 | 55 |  |
| 2y | SF | 34.6 |  |  | 47.4 | 281 | 16 | 81 | 231.09 | 2.2 | 5.5 | 38.7 | 50.5 | 2.72362 | 55 |  |
| 2y | CM | 29 |  |  | 54.2 | 196 | 16 | 78 | 120.91 | 3.2 | 7.2 | 32 | 47.8 | 2.57658 | 59 |  |
| 2y | SF | 26 |  |  | 42.2 | 220 | 14 | 90 | 673.63 | 1.5 | 4.1 | 55.3 | 50.4 | 2.20824 | 59 |  |
| 4y | SF | 30.7 |  |  | 52.8 | 175 | 17 | 92 | 133.1 | 1.6 | 5.2 | 54.2 | 44.7 | 1.31379 | 47 |  |
| 2y | IF | 28 |  |  | 52.3 | 300 | 17 | 92 | <50 | 2.2 | 1.2 | 72.8 | 62.3 | 3.81515 | 48 |  |
| 4y | CM | 34.8 |  |  | 49.7 | 237 | 17 | 78 | 70.35 | 2.8 | 3.8 | 50.9 | 50.1 | 2.42138 | 109 |  |
| 6y | SF | 47.5 |  |  | 50 | 189 | 14 | 83 | 2199.84 | 2 | 2.3 | 60.6 | 61 | 3.8796 | 55 |  |
| 1y | CM | 33.4 |  |  | 54.5 | 178 | 17 | 77 | 117.3 | 1.1 | 0.8 | 80.1 | 58.9 | 2.93787 | 121 |  |
| 2y | CM | 26 |  |  | 52.2 | 220 | 16 | 85 | <50 | 1.6 | 4.8 | 63.8 | 46.1 | 1.31045 | 60 |  |
| 3y | SF | 35 |  |  | 51.7 | 163 | 17 | 79 | <50 | 2.2 | 6.6 | 41 | 42 | 1.27156 | 74 |  |
| 5y | CM | 25 |  |  | 54.5 | 151 | 14 | 75 | 260.27 | 1.2 | 5.6 | 63.3 | 50.2 | 1.95933 | 50 |  |
| 2y | CM | 23.9 |  |  | 55.6 | 157 | 14 | 89 | <50 | 2.4 | 7.7 | 45.1 | 41.1 | 1.05846 | 120 |  |
| 3y | SF | 34 |  |  | 47.9 | 202 | 12 | 72 | 574.99 | 2.8 | 5.3 | 39.4 | 50.7 | 2.81163 | 110 |  |
| 1y | CM | 31.8 |  |  | 51.4 | 156 | 12 | 72 | 153.82 | 3.2 | 3.3 | 54.5 | 56 | 3.35555 | 71 |  |
| 4y | CM | 50.1 |  |  | 45.6 | 182 | 13 | 81 | 110.27 | 1.2 | 1.8 | 73.4 | 58.1 | 2.98841 | 54 |  |
| 3y | CM | 38 |  |  | 47.9 | 228 | 15 | 85 | 82.41 | 1.4 | 4.8 | 60 | 44.2 | 1.06304 | 44 |  |
| 1y | SF | 17 |  |  | 53.9 | 240 | 14 | 95 | 236.06 | 2.7 | 2.1 | 62 | 61.4 | 3.99611 | 44 |  |
| 2y | CM | 40 |  |  | 46.5 | 229 | 12 | 77 | 190.39 | 1.2 | 3.2 | 69 | 57.9 | 3.07423 | 66 |  |
| 6y | CM | 41 |  |  | 46.3 | 200 | 14 | 83 | 895.63 | 0.7 | 0.8 | 81.7 | 62.5 | 3.44603 | 45 |  |
| 6y | SF | 27 |  |  | 43.7 | 217 | 16 | 75 | 109.11 | 2.8 | 1.7 | 65.7 | 57.6 | 3.28663 | 42 |  |
| 13y | CM | 5.4 | MMVD ACVIM stage Cc, Pulmonary hypertension |  | 57.7 | 562 | 14 | 101 | 9936.97 | 5 | 2.7 | 43.6 | 70.1 | 6.16713 | 111 |  |
| 14y | CM | 7.2 | Hepatic mass, Pancreatitis |  | 22.6 | 310 | 13 | 98 | 4546.38 | 1.6 | 0.8 | 79.1 | 78.7 | 6.30022 | 87 |  |
| 13y | IF | 3.93 | Pyometra |  | 30.4 | 570 | 16 | 119 | 1141.1 | 4.1 | 0.9 | 76.8 | 82.2 | 7.24257 | 76 |  |
| 7y | IF | 3.85 | Pneumonia |  | 27.5 | 476 | 12 | 102 | 1780.21 | 3 | 0.9 | 78.8 | 85.1 | 7.53935 | 70 |  |
| 7y | IF | 9.4 | Enteritis, Primary MDS, Hypothyroidism |  | 17.6 | 328 | 15 | 82 | 127.7 | 5.2 | 2.1 | 59.7 | 83 | 7.93309 | 91 |  |
| 5y | IM | 10.9 | SLE, Erythema multiforme |  | 24.1 | 258 | 14 | 101 | 5276.99 | 6.4 | 1.8 | 69.4 | 79 | 7.1818 | 58 |  |
| 11y | CM | 4.4 | Idiopathic portal hypertension | Liver biopsy, Splenectomy | 28.9 | 450 | 11 | 93 | over | 2.6 | 1 | 77.5 | 77.9 | 6.33092 | 57 |  |
| 12y | CM | 5.5 | S3 fracture, coccygeal luxation | Osteosynthesis | 32.6 | 407 | 16 | 101 | 520.09 | 2.9 | 0.8 | 78 | 79.3 | 6.58554 | 55 |  |
| 12y | SF | 6.2 | Ophthal morrhexis | OD enucleation | 35.3 | 339 | 14 | 104 | 618.85 | 4.8 | 2 | 63.9 | 78.5 | 7.03712 | 50 |  |
| 12y | CM | 3.22 | Perineal hernia | Perineal herniorrhaphy, Colopexy, Cystopexy | 41.8 | 308 | 16 | 112 | 450.46 | 2.8 | 1.1 | 74 | 76.9 | 6.27523 | 62 |  |
| 13y | CM | 15.5 | L4-5 IVDD | Hemilaminectomy | 44.3 | 323 | 14 | 109 | 927.58 | 2.1 | 0.8 | 78.1 | 79.6 | 6.53462 | 99 |  |
| 14y | CM | 4 | MMVD stage Cc, tracheal collapse |  | 28 | 361 | 15 | 86 | under | 1.6 | 0.8 | 80.3 | 74.4 | 5.55965 | 64 |  |
| 9y | CM | 6.27 | Pancreatitis |  | 50.1 | 301 | 14 | 83 | 286.86 | 2 | 1.6 | 70.4 | 71.8 | 5.42438 | 83 |  |
| 4y | SF | 12.5 | Atopic dermatitis |  | 43 | 295 | 13 | 88 | 282.1 | 2.2 | 1 | 75 | 74.1 | 5.71319 | 54 |  |
| 4y | CM | 4.6 | Osteomyelitis |  | 37.2 | 179 | 14 | 88 | 1415.13 | 2.7 | 1.1 | 72.9 | 71.4 | 5.37922 | 41 |  |
| 10y | SF | 4.37 | Urolithiasis | Cystotomy | 41.2 | 521 | 14 | 107 | 146.33 | 4.8 | 1.5 | 69.6 | 77.8 | 6.7793 | 109 |  |
| 12y | SF | 4.67 | Addison’s disease, Enteritis |  | 34.5 | 354 | 12 | 90 | 202.27 | 3.2 | 1 | 75.9 | 79.6 | 6.72445 | 94 |  |
| 12y | SF | 3.5 | gallbladder mucocele | Cholecystectomy | 26.2 | 100 | 13 | 107 | 4339.51 | 3.2 | 0.9 | 71.7 | 81.1 | 7.073 | 244 |  |
| 12y | CM | 4.9 | gallbladder mucocele | Cholecystectomy | 29.8 | 181 | 13 | 105 | 86.45 | 2.5 | 0.9 | 76.1 | 76.1 | 6.05357 | 90 |  |
| 8y | CM | 10.9 | AKI stage III, Acute pancreatitis |  | 34.8 | 443 | 13 | 96 | 1021.71 | 3.3 | 1.1 | 65.9 | 81.4 | 7.27654 | 76 |  |
| 9y | IF | 4.1 | Pyelonephritis, bacteria cystitis |  | 40.3 | 720 | 12 | 89 | 719.63 | 4 | 1.4 | 68.4 | 70.4 | 5.48444 | 64 |  |
| 11y | CM | 7.88 | Hyperadrenocorticism, calcinosis cutis |  | 34.9 | 591 | 14 | 100 | 230.02 | 2.8 | 0.8 | 76.3 | 81.5 | 6.97834 | 48 |  |
| 10y | SF | 22 | liposarcoma, peritonitis |  | 38.6 | 394 | 14 | 80 | 1615.68 | 3.2 | 0.8 | 72.9 | 77.5 | 6.44736 | 49 |  |
| 2y | SF | 12.5 | IMHA |  | 29.8 | 192 | 13 | 78 | 8518.45 | 2.2 | 0.8 | 82.6 | 84.2 | 7.19974 | 66 |  |
| 11y | SF | 2.38 | Renal HAS, Pancreatitis |  | 37 | 318 | 11 | 102 | 705.51 | 4.7 | 0.8 | 77.1 | 83.9 | 7.58939 | 118 |  |
| 15y | SF | 9.5 | MMVD ACVIM stage B1 w/ pCTR, Soft tissue sarcoma (Rt. hindlimb) |  | 30.1 | 381 | 14 | 90 | 693.81 | 3.6 | 1 | 76.4 | 81.1 | 7.00973 | 66 |  |
| 10y | CM | 2.25 | Salmonellosis |  | 35.8 | 584 | 13 | 105 | 694.63 | 2.5 | 0.8 | 82.7 | 79.7 | 6.48939 | 56 |  |
| 7y | CM | 18 | Perianal fistula |  | 37.7 | 177 | 14 | 92 | 1049.15 | 2.7 | 1.1 | 74.7 | 75.2 | 5.96474 | 49 |  |
| 11y | CM | 6.7 | IMT |  | 31.4 | 329 | 11 | 92 | 3889.9 | 3.2 | 1.1 | 74 | 71.7 | 5.46371 | 55 |  |
| 12y | CM | 3.92 | MMVD ACVIM stage B1, Hyperadrenocorticism |  | 40.4 | 548 | 13 | 77 | 467.33 | 3.2 | 1.4 | 68.9 | 70.8 | 5.44043 | 79 |  |

SF; spayed female, IF; intact female, CM; castrated male, IM; intact male, HCT; hematocrit, PLT; platelet count, PT; prothrombin time, aPTT; activated partial thromboplastin time, R; reaction time, K; coagulation time, α; alpha angle, MA; maximum amplitude, CI; coagulation index, MMVD; myxomatous mitral valve disease, MDS; myelodysplastic syndrome, SLE; systemic lupus erythematosus, IVDD; intervertebral disc disease, AKI; acute kidney injury, IMHA; immune-mediated hemolytic anemia, HAS; hemangiosarcoma, IMT; immune-mediated thrombocytopenia, pCTR; partial chordae tendineae rupture.
